# Distinct Trends of DNA Methylation Patterning in the Innate and Adaptive Immune Systems

**DOI:** 10.1016/j.celrep.2016.10.054

**Published:** 2016-11-15

**Authors:** Ronald P. Schuyler, Angelika Merkel, Emanuele Raineri, Lucia Altucci, Edo Vellenga, Joost H.A. Martens, Farzin Pourfarzad, Taco W. Kuijpers, Frances Burden, Samantha Farrow, Kate Downes, Willem H. Ouwehand, Laura Clarke, Avik Datta, Ernesto Lowy, Paul Flicek, Mattia Frontini, Hendrik G. Stunnenberg, José I. Martín-Subero, Ivo Gut, Simon Heath

**Affiliations:** 1 CNAG-CRG, Centre for Genomic Regulation (CRG), Barcelona Institute of Science and Technology (BIST), Baldiri i Reixac 4, Barcelona 08028, Spain; 2 Universitat Pompeu Fabra (UPF), Barcelona 08002, Spain; 3 Dipartimento di Biochimica Biofisica e Patologia Generale, Seconda Università degli Studi di Napoli, Vico Luigi de Crecchio 7, Napoli 80138, Italy; 4 Department of Hematology, University of Groningen and University Medical Center Groningen, PO Box 30001, 9700 RB Groningen, the Netherlands; 5 Department of Molecular Biology, Radboud University, Faculty of Science, Nijmegen Centre for Molecular Life Sciences, 6500 HB Nijmegen, the Netherlands; 6 Department of Blood Cell Research, Sanquin Research and Landsteiner Laboratory, Academic Medical Center, University of Amsterdam, Plesmanlaan 125, 1066 CX Amsterdam, the Netherlands; 7 Emma Children’s Hospital, Academic Medical Center, University of Amsterdam, Meibergdreef 9, 1105 AZ Amsterdam, the Netherlands; 8 Department of Haematology, University of Cambridge, Cambridge Biomedical Campus, Long Road, CB2 0PT Cambridge, UK; 9 National Health Service (NHS) Blood and Transplant, Cambridge Biomedical Campus, Long Road, CB2 0PT Cambridge, UK; 10 British Heart Foundation Centre of Excellence, University of Cambridge, Cambridge Biomedical Campus, Long Road, CB2 0QQ Cambridge, UK; 11 Department of Human Genetics, The Wellcome Trust Sanger Institute, Wellcome Trust Genome Campus, Hinxton, CB10 1HH Cambridge, UK; 12 European Molecular Biology Laboratory, European Bioinformatics Institute, Wellcome Genome Campus, Hinxton, CB10 1SD Cambridge, UK; 13 Department of Anatomic Pathology, Pharmacology and Microbiology, University of Barcelona, Institut d’Investigacions Biomédiques August Pi i Sunyer (IDIBAPS), Barcelona 08036, Spain

## Abstract

DNA methylation and the localization and post-translational modification of nucleosomes are interdependent factors that contribute to the generation of distinct phenotypes from genetically identical cells. With 112 whole-genome bisulfite sequencing datasets from the BLUEPRINT Epigenome Project, we analyzed the global development of DNA methylation patterns during lineage commitment and maturation of a range of immune system effector cells and the cancers that arise from them. We show clear trends in methylation patterns that are distinct in the innate and adaptive arms of the human immune system, both globally and in relation to consistently positioned nucleosomes. Most notable are a progressive loss of methylation in developing lymphocytes and the consistent occurrence of non-CG methylation in specific cell types. Cancer samples from the two lineages are further polarized, suggesting the involvement of distinct lineage-specific epigenetic mechanisms. We anticipate broad utility for this resource as a basis for further comparative epigenetic analyses.

## Introduction

Different cell types stably express distinct phenotypes despite sharing an identical underlying genotype. Chemical modifications to DNA and associated histones allow genetically identical cells to exhibit radically different behavior and morphology by shaping gene expression programs and cellular responses to stimuli (Luperchio et al., 2014; Peric-Hupkes et al., 2010; Shen and Laird, 2013).

Well-defined differentiation programs in the hematopoietic system provide an ideal model to investigate the mechanisms regulating cell identity. Lineage choice (myeloid or lymphoid) followed by further specialization and stable states of quiescence, activation, or long-term memory offer an established framework for studying epigenetic processes (Kondilis-Mangum and Wade, 2013; Russ et al., 2013). Previous studies examining such modifications have contributed substantial insights into immune system function and dysfunction (Cedar and Bergman, 2011; Farh et al., 2015).

DNA methylation is a ubiquitous epigenetic mark that is written directly onto DNA as the addition of a methyl group to a cytosine residue. Most DNA methylation occurs at cytosines followed by a guanine residue (CG dinucleotides), and the bulk of CGs genome-wide are methylated (Lister et al., 2009). Large-scale methylation patterns distinguish cell types (Hodges et al., 2011), and stable control of the methylome increases the stability of a given cell state (Raynal et al., 2012). The spatial organization and epigenetic patterning of the genome both deteriorate progressively over the lifetime of an organism (Sinclair and Oberdoerffer, 2009) and are often markedly disorganized in cancer and human genetic disorders of premature aging (Heyn et al., 2013; Reddy and Feinberg, 2013). Epigenetic modifiers, such as the DNA methyltransferase *DNMT3A* and the demethylase *TET2*, are commonly mutated in cancer (Jasielec et al., 2014; Shih et al., 2012). Many cancer subtypes have an identifiable methylation signature (Shen and Laird, 2013), and methylation patterns of human cells approaching senescence (Cruickshanks et al., 2013) and of long-lived immunological memory cells and plasma cells often begin to resemble those commonly observed in cancer and in immortalized cell lines (Kulis et al., 2012, 2015). In addition, recent studies have documented the ability of pathogens to directly and specifically modulate host epigenomes to dampen the immune response and enhance their own survival (Jose et al., 2016; Pacis et al., 2015; Sharma et al., 2015).

Two key components of genomic organization that are reflected in, and affected by, methylation are the DNA-binding protein CTCF (Ong and Corces, 2014; Wang et al., 2012) and nucleosomes, the basic structural units of chromatin (Mueller-Planitz et al., 2013). Across the vast majority of the human genome, nucleosome positions are not consistent between cells, even within homogeneous cell populations, as evidenced by the generally fuzzy picture produced by genome-wide nucleosome footprinting studies (Gaffney et al., 2012; Valouev et al., 2011). Exceptions occur in the 1 or 2 kb of DNA surrounding occupied CTCF-binding sites and in the ∼0.5 kb immediately downstream of transcription start sites, where regular and consistent nucleosome spacing is evident as periodic peaks and valleys in nuclease-digested read counts (Fu et al., 2008).

Numerous experimental studies support the conclusion that nucleosomes protect occupied DNA from methylation, directing it preferentially to adjacent linker regions (Felle et al., 2011; Takeshima et al., 2008; Zhang et al., 2010), and that methylation disfavors nucleosome occupancy (Jimenez-Useche et al., 2013; Pérez et al., 2012; Portela et al., 2013). Those results are consistent with others directly correlating low nucleosome occupancy with high methylation levels within individual DNA molecules (Kelly et al., 2012) and across species (Huff and Zilberman, 2014). However, counterexamples exist, describing higher methylation levels associated with nucleosome occupancy genome-wide (Chodavarapu et al., 2010; Jin et al., 2012). Conflicting reports may be attributable to different levels of data resolution or quality or to the choice of scale for the analysis. Areas of phased, consistently positioned nucleosomes surrounding CTCF-binding sites provide an opportunity to investigate the relationship between DNA methylation and nucleosome occupancy at these structurally important sites across a range of cell types.

To investigate the epigenetic mechanisms guiding cell identity and to provide a resource for the research community, the BLUEPRINT Epigenome Project has produced more than 200 whole-genome bisulfite sequencing (WGBS) DNA methylation maps, as well as genome-wide maps for six histone modifications, DNase-I hypersensitivity, and RNA expression for the large majority of these samples, covering the development of the major branches of the human immune system and several cancers that arise from them. Here, we integrated WGBS methylation maps for each of 112 samples from BLUEPRINT with CTCF-binding data from ENCODE and nucleosome occupancy derived from micrococcal nuclease digestion sequencing (MNase-seq) and chromatin immunoprecipitation sequencing (ChIP-seq) data from BLUEPRINT samples and ENCODE cell lines.

We follow the development and activation of monocytes/macrophages and neutrophils in the myeloid lineage, and T and B lymphocytes in the lymphoid branch, and demonstrate consistent lineage-specific differences in the usage of DNA methylation globally and in relation to consistently positioned nucleosomes. We note progressive methylation shifts with differentiation and methylation trends in cancer samples that become more distinct between the two lineages. We also describe two distinct forms of apparent non-CG methylation: diffuse, globally high levels in naive T cells and uncommitted hematopoietic progenitor cells and dense exon-specific non-conversion in a few samples. All BLUEPRINT data are made available via http://www. blueprint-epigenome.eu.

## Results

### Global Methylation Trends

**Figure 1:**
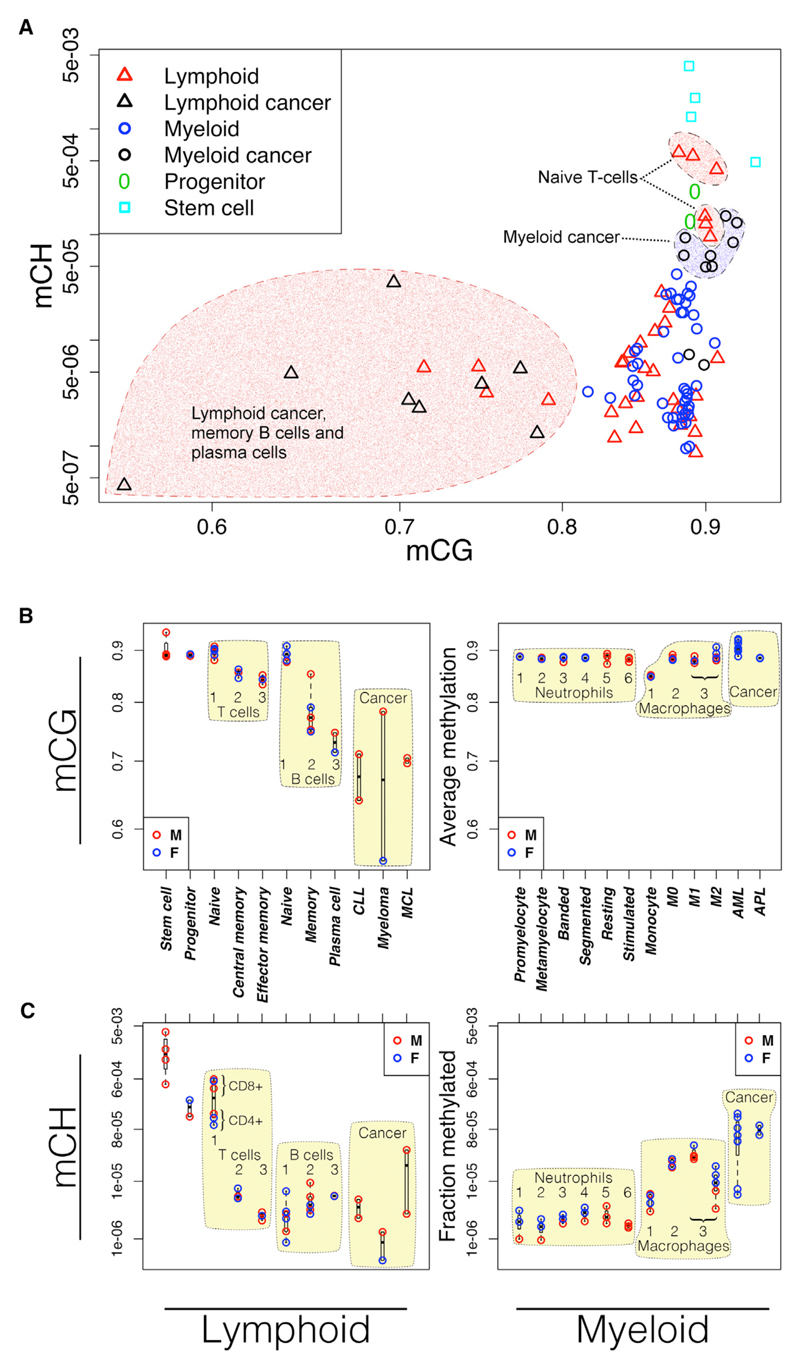
Genome-wide DNA Methylation Trends in Cells of the Lymphoid and Myeloid Lineages (A) Methylation in CH and CG contexts in lymphoid (triangles) and myeloid cells (circles). Long-lived lymphoid cells have lower CG methylation. Naive T cells have higher levels of non-CG methylation. Most myeloid-derived cancers gain non-CG methylation. (B) CG methylation. Developing lymphocytes lose methylation (left). Cells of the myeloid lineage are relatively constant or show small gains in methylation (right). (C) Fraction of non-CG cytosines methylated. Fraction methylated is computed as the number of significantly methylated non-CG cytosines divided by the total number of non-CG cytosines read with adequate coverage to make a methylation call, as described in Experimental Procedures. Stem cell: HUES64 cell line; progenitor: CD34^+^, including stem cells, multipotent progenitors, and common lymphoid progenitors. Numbers indicate developmental order relative to the indicated cell type. Blue, female; red, male.

Trends in global average CG methylation are markedly distinct between the lymphoid and myeloid lineages (Figure 1). Following commitment to the myeloid lineage, global average CG methylation levels remain high and relatively stable throughout differentiation and activation. Slightly higher methylation levels are seen in macrophages relative to monocytes (t test; monocytes versus M0: p = 2 × 10^−4^; M1: p = 1 × 10^−3^; M2: p = 8 × 10^−4^), but the difference is small in comparison to the large differences among lymphocytes.

In both T and B lymphocytes, global methylation drops progressively with sequential stages of differentiation, resulting in the lowest genome-wide methylation levels in long-lived memory B cells and plasma cells, lymphoid cancers, and experimentally transformed B cells (Figures 1A and 1B). The dynamics of demethylation in T and B lymphocytes compared with the relatively constant levels across the myeloid lineage suggests substantially different usage of DNA methylation between the two lineages.

### Non-CG Methylation

**Figure 2:**
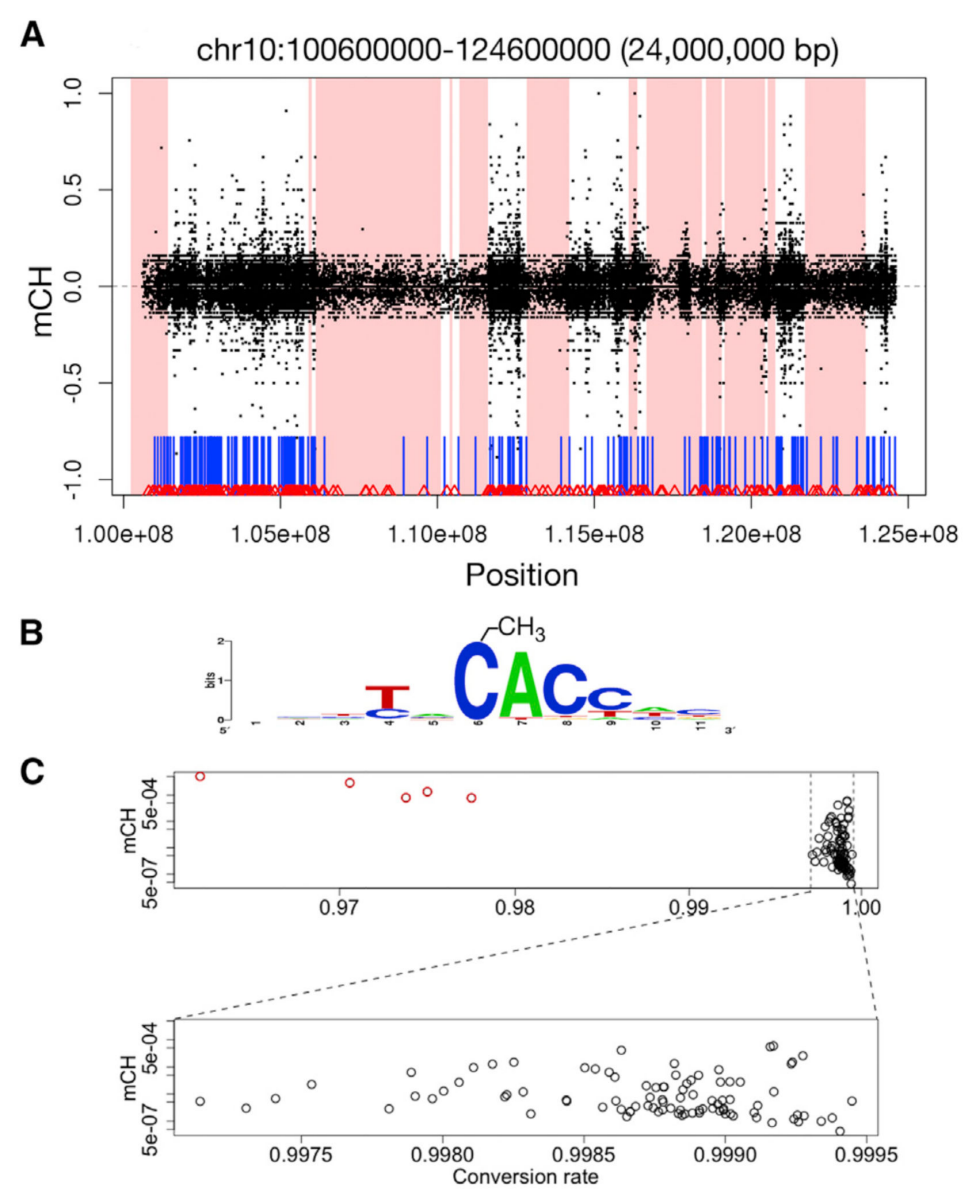
Non-CG Methylation (A) Example of mCH exclusion from lamina-associated domains (shaded areas) in a naive T cell, chr10:100,600,000–124,600,000. x axis, genomic location; y axis, non-CG methylation level (negative values, minus strand; positive values, plus strand). CpG islands (vertical blue bars) and CTCF-binding sites (red triangles) are shown. (B) Information content in the sequence context of mCH in naive T cells. The methylated cytosine is at position 6. (C) Conversion rate and mCH. After exclusion of under-converted outliers (red, top plot), the fraction of methylated non-CG cytosines is not influenced by conversion rate (bottom expanded plot), as determined using unmethylated spiked-in bacteriophage DNA. mCH is computed as the fraction of non-converted non-CG cytosines.

Methylation outside of a CG dinucleotide context (mCH, where H = {A,C,T}) is prevalent in stem cells and neurons but was believed to be absent from other cell types (Ziller et al., 2011; Lister et al., 2013). The non-random distribution of mCH in a range of tissues is now generally accepted (He and Ecker, 2015; Schultz et al., 2015). Most BLUEPRINT samples contain only background levels of mCH, likely reflecting very low levels of incomplete sodium bisulfite conversion of unmethylated cytosines. We excluded five under-converted samples, and all remaining samples had conversion rates greater than 99.7%. Following exclusion of those samples below this stringent threshold, measured mCH levels are not influenced by conversion rate (Figure 2C).

We found high levels of mCH in all naive T cell samples (three CD4^+^ and three CD8^+^) and in uncommitted hematopoietic progenitor cells (UHPs) (Figure 1C). The fraction of non-CG cytosines that are methylated in these samples is significantly higher than in other cell types from either lineage (t test; p = 5.7 × 10^−3^) and is more similar to levels seen in stem cells. This widespread mCH disappears with subsequent stages of T cell development. Macrophage samples (M0, M1, and M2) also have elevated levels of mCH relative to most committed cell types but do not reach the levels seen in progenitors and naive T cells. Leukemias derived from the myeloid lineage (acute promyelocytic leukemia [APL] and acute myeloid leukemia [AML]) appear to consistently gain considerable mCH (p = 2.8 × 10^−3^; t test relative to all other myeloid samples). In lymphoid-derived neoplasms (mantle cell lymphoma [MCL], chronic lymphocytic leukemia [CLL], and multiple myeloma [MM]), mCH appears more varied (Figure 1C). Cancer sample source materials are described in Table S1.

Diffuse mCH is found overwhelmingly in a CA context (>90%), with CAC accounting for 72% of methylated non-CG cytosines in naive T cells (Figure 2B). CAC is the predominant context of mCH in neurons, whereas CAG is more common in the H1 stem cell line (Lister et al., 2009; Schultz et al., 2015; Varley et al., 2013).

In all hematopoietic samples where it occurs at appreciable levels, non-CG methylation is enriched in genes (exons and/or introns), to varying degrees. At the scale of whole chromosomes, the most distinctive feature of the high levels of mCH is their exclusion from lamina-associated domains (LADs) (Figure 2A; p < 10^−6^). Because LADs cover 1/3 of the genome and are gene poor, mCH exclusion from these regions may contribute to the observed enrichment in genes.

**Figure 3:**
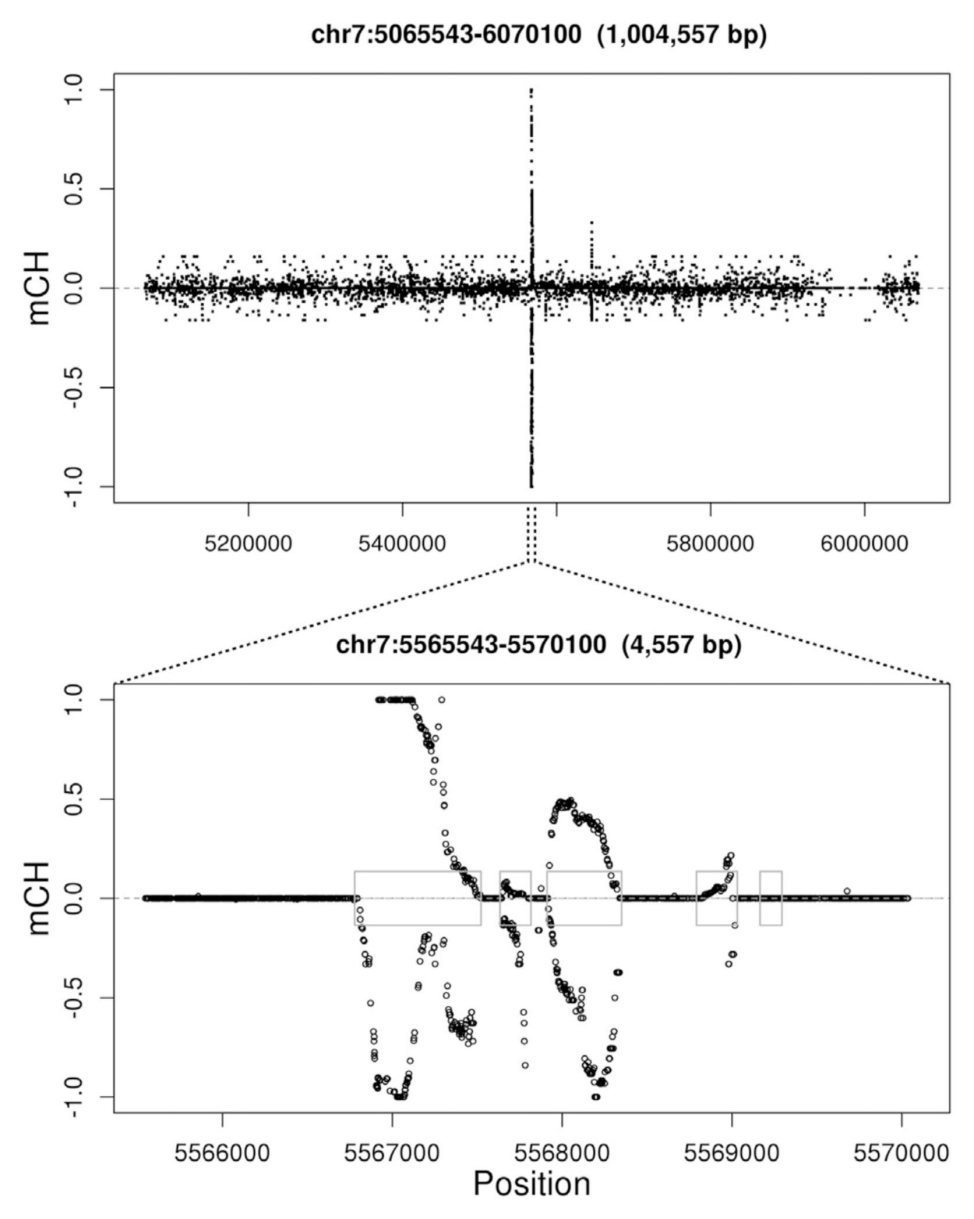
Exon-Specific CH Non-conversion Two plots at the same genomic location (x axis) at different resolutions. (Top plot) Spikes rise above background mCH levels. (Bottom plot) Spikes are specific to exons (blue boxes). y axis, fraction of non-converted reads at non-CG cytosines. Negative values indicate cytosines on the minus strand. Only positions with six or more reads informative for methylation status are shown. See also Figure S1.

In addition to the diffuse mCH seen in all uncommitted progenitors and naive T cells, we also noticed short contiguous regions of non-CG cytosines protected from bisulfite conversion that are localized precisely to exons in one of the two MCL samples and in two normal samples (Figure 3). These spikes in non-converted non-CG cytosines occur in three and sixteen exons in a neutrophil and monocyte sample, respectively, and in more than 400 exons and pseudogenes in the MCL sample. As shown in the examples in Figure S1, the putative mCH spikes occur at areas of transition in CG methylation levels. Although striking, we note that these mCH spikes are not observed in other samples of the same cell types, making it impossible to draw robust conclusions with the limited examples currently available.

### DNA Methylation Reflects Nucleosome Positioning

**Figure 4:**
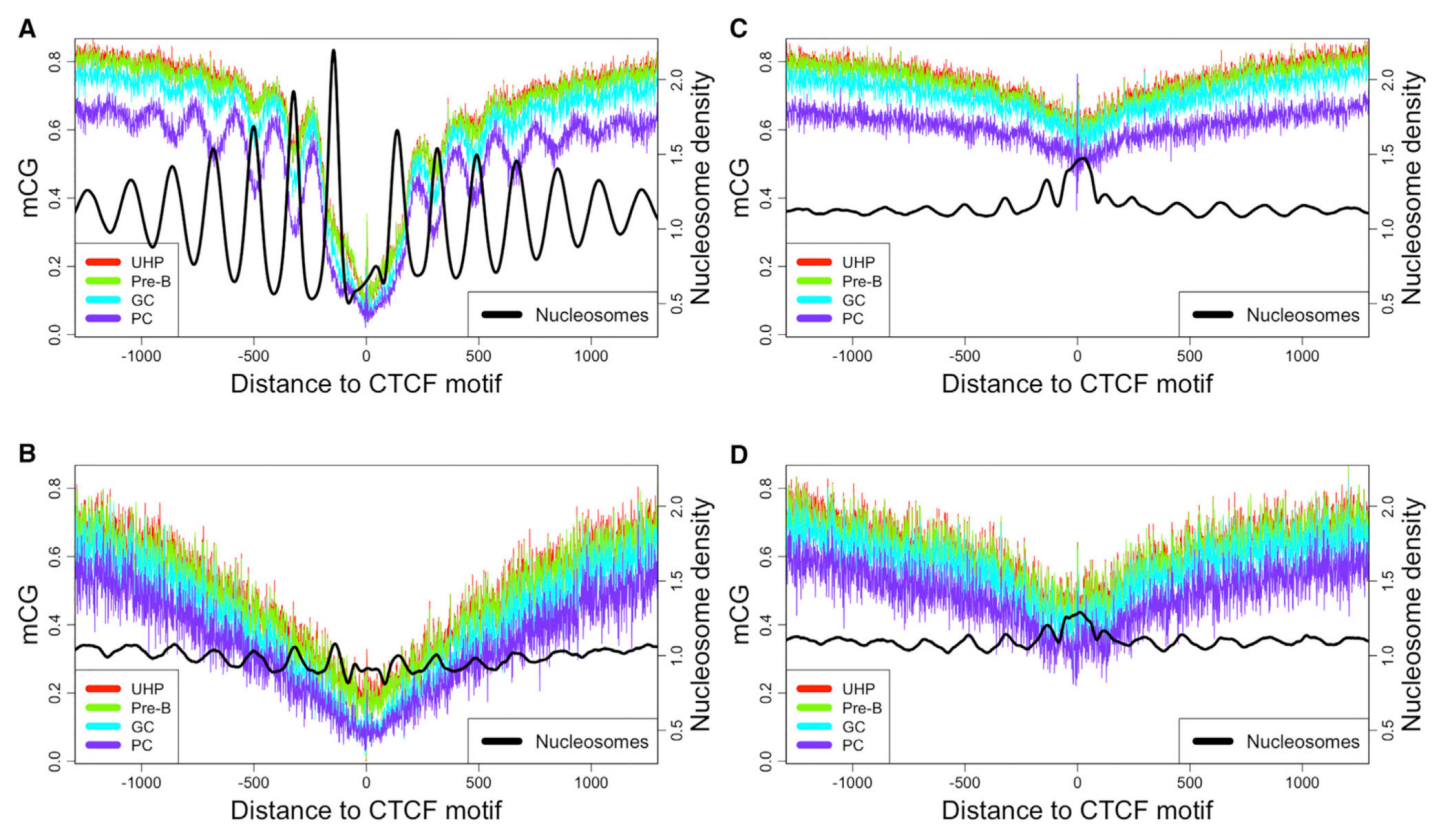
DNA Methylation Levels at Successive Stages of B Lymphocyte Development Aggregated across Subsets of CTCF-Binding Sites Development occurs in the order indicated in the legend, with UHP being the least differentiated cell type. (A) Methylation surrounding 23,710 constitutively occupied CTCF-binding sites shows oscillations that increase in amplitude as lymphocytes develop. (B–D) CTCF-binding sites specifically occupied in (B) lymphoblastoid cell line, (C) stem cell line (unoccupied in B cells), and (D) skin cell line (unoccupied in B cells). GC, germinal center B cell; PC, plasma cell; UHP, uncommitted hematopoietic progenitor. See also Figures S2 and S3.

In all samples, DNA methylation levels near CTCF display an oscillatory pattern with a period of approximately 175 bp, consistent with one unit of nucleosome-occupied DNA plus one linker segment (Fu et al., 2008). We compared methylation levels with experimentally determined nucleosome occupancy data from previously published studies using MNase-seq (Valouev et al., 2011) and nucleosome positions derived from ChIP-seq experiments using BLUEPRINT samples (Mammana et al., 2013). Methylation and nucleosome occupancy oscillate in counterphase, with peaks in methylation corresponding to valleys in nucleosome density (Figure 4A). In contrast with other results (Teif et al., 2014), the bias for increased methylation between nucleosomes is consistent both within and outside of CpG islands (Figure S2), although levels of both methylation and nucleosome occupancy are generally lower within CpG islands.

We separated CTCF-binding sites into those which are constitutively occupied (cCTCF), occupied only in specific cell types, or unoccupied, as in Li et al. (2013), revealing that oscillating methylation levels are driven by the constitutively occupied sites (Figure 4). Although occupied cell-type-specific binding sites display a valley in average methylation levels, most have little or no discernible oscillatory pattern (Figure 4B). Stem cell lines (H1 and H9) are the exception, where methylation near the stem-cell-specific CTCF sites also displays the clear oscillatory pattern characteristic of cCTCF sites (Figure S3C). Unoccupied CTCF sites display minimal oscillation and a much less pronounced dip in methylation immediately surrounding the binding site (Figures 4C and 4D).

**Figure 5:**
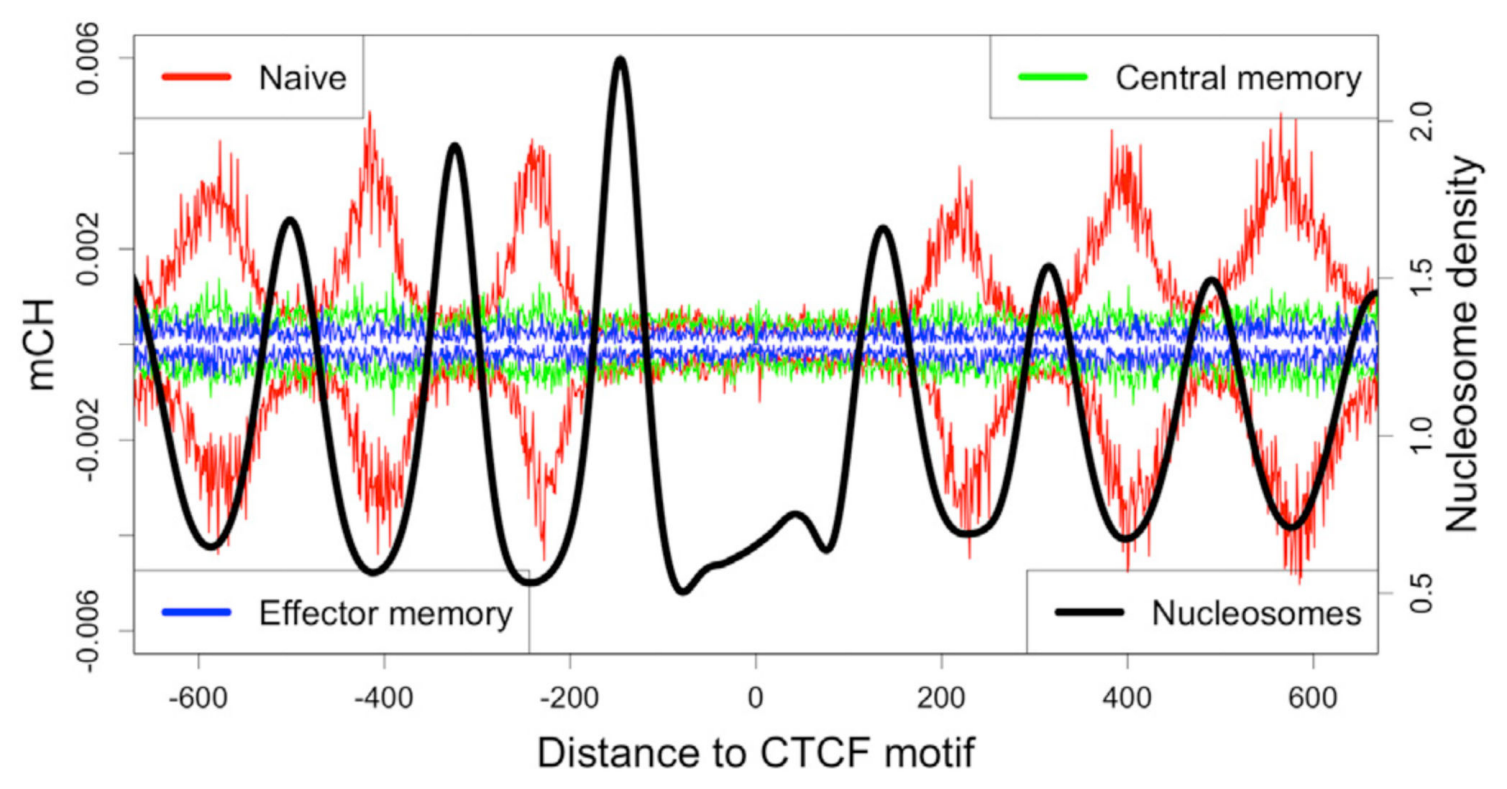
mCH in T Lymphocytes mCH occurs between nucleosomes (black) in naïve T lymphocytes (red), but not memory T lymphocytes (green and blue). Positive and negative methylation values correspond to cytosines on the plus and minus strand, respectively. Values are aggregated across 23,710 constitutively occupied CTCF sites.

In each sample where it occurs, mCH follows a similar nucleosome-influenced spatial enrichment pattern as CG methylation near cCTCF, but the bias for mCH toward linker regions is even more pronounced than for CG methylation, with almost complete exclusion from nucleosome-occupied DNA (Figure 5). Aggregate methylation levels are similar on the plus and minus strands relative to the orientation of the CTCF motif.

In line with our methylation results, the oscillation of the nucleosome positioning signal at cell-type-specific CTCF sites is much flatter than the strong alternating enrichment and depletion characteristic of the cCTCF sites. We observe regular positioning surrounding cCTCF sites using nucleosome occupancy data derived from MNase digestion in either a lymphoid or myeloid cell line; we further confirm this observation using nucleosome positions derived from the integration of ChIP-seq data for six histone modification marks (Mammana et al., 2013) obtained from the same sorted primary cell samples used for the DNA methylation analysis (Figure S4). We conclude that both nucleosomes and DNA methylation are stably configured at cCTCF across cell types, but not at unoccupied sites (Chen et al., 2012).

### Development of Nucleosome-Influenced Methylation Patterns

Visual inspection of Figure 4A reveals a progressive increase in the amplitude of oscillation in the methylation signal, corresponding to B lymphocyte development. To facilitate comparison across many samples, we define an objective measure of nucleosome influence based on the difference in methylation levels between the constitutively nucleosome-occupied DNA and the adjacent linker DNA (Figure S5; Experimental Procedures).

**Figure 6:**
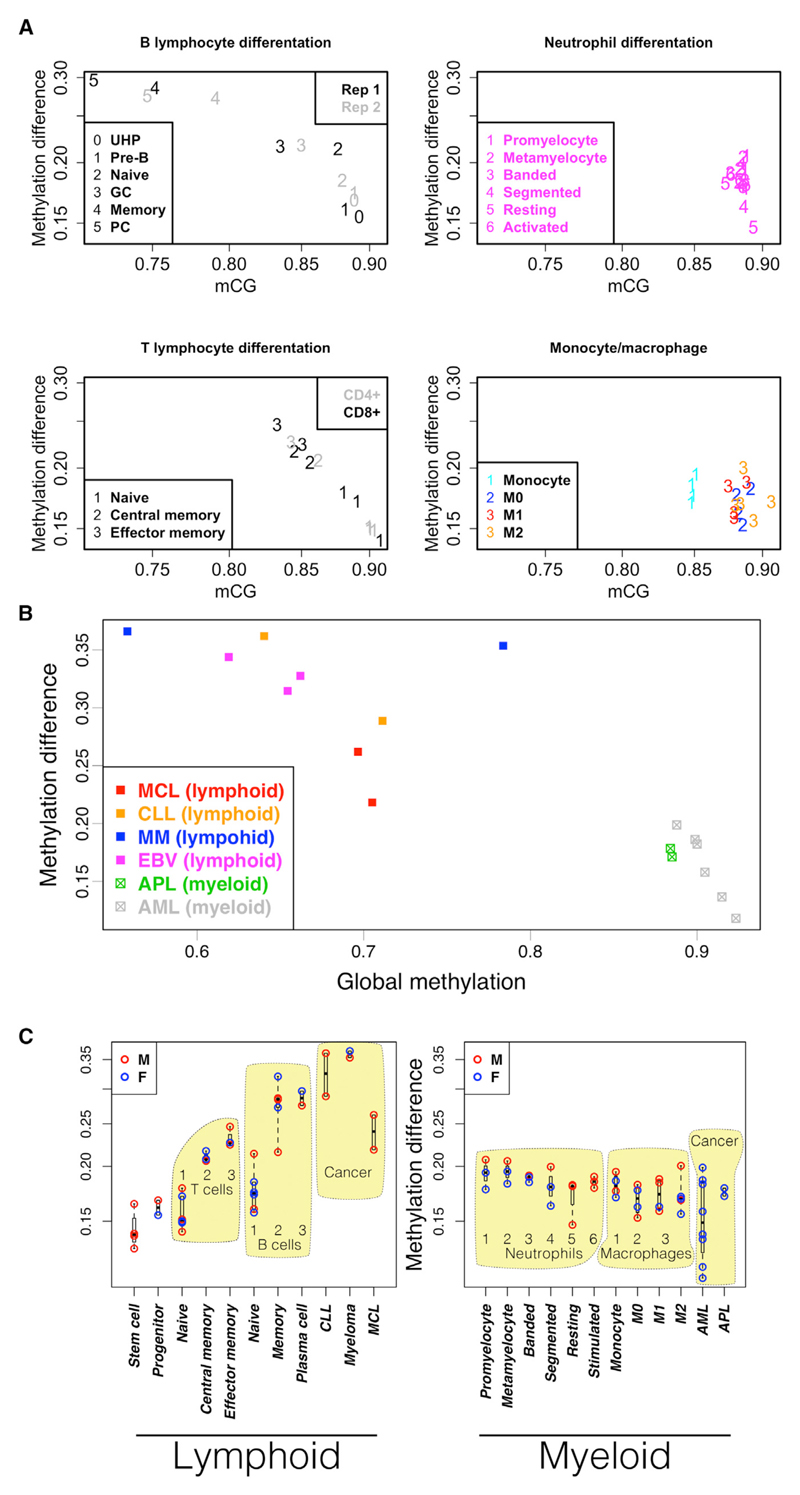
Nucleosome Influence and DNA Methylation in Lymphoid and Myeloid Development Nucleosome influence (y axis) is defined as the difference in methylation between nucleosome-occupied DNA and the adjacent linker DNA at cCTCF. Data are aggregated across 23,710 constitutively occupied CTCF sites. (A) Normal development in two cell types from each lineage. Two replicates of B lymphocyte development: 0, UHP; 1, pre-B; 2, naive B cell; 3, germinal center B cell; 4, memory B cell; 5, plasma cell. T lymphocytes: CD4^+^ (gray) and CD8^+^ (black). 1, naive T cell; 2, central memory T cell; 3, effector memory T cell. Neutrophil development: 1, promyelocyte; 2, metamyelocyte, 3, banded neutrophil; 4, segmented neutrophil; 5, resting neutrophil; 6, GTX-activated neutrophil. Monocyte/macrophage development: 1, monocyte; 2, resting macrophage (M0); 3, activated macrophages (red:M1; orange:M2). (B) Neoplasms derived from lymphoid and myeloid lineages. (C) Comparison across cell types. Numbers indicate developmental order as in (A). See also Figure S5.

In both B and T lymphocytes, the oscillatory methylation pattern at cCTCF sites becomes more pronounced with successive stages of development while global average methylation levels decline (Figure 6A). Differential methylation analysis over five stages of B lymphocyte differentiation and activation reveals enrichment of significant methylation gains at linker DNA and losses at nucleosome-associated DNA (Figure S6). This progressive shift of methylation from nucleosomes to linkers in longer-lived lymphocytes is consistent with the idea of a reciprocal methylation/nucleosome relationship that builds over time.

In contrast to the methylation trends seen in developing lymphocytes, the oscillating methylation pattern near cCTCF in the myeloid lineage is less varied and shows no consistent trend (Figures 6A and 6C).

Neoplastic transformation results in increased lineage-specific differences in linker methylation bias (Figures 6B and 6C). Cancers derived from the lymphoid lineage reflect a continued shift of methylation from nucleosomes to linkers, in both experimentally induced EBV (Epstein-Barr virus) immortalization and primary lymphoid tumor samples (MCL, CLL, and MM). Myeloid-derived cancer samples (APL and AML), however, display a methylation oscillation amplitude that is either comparable to that of normal samples from that lineage or substantially decreased, corresponding to increased global methylation levels.

## Discussion

DNA methylation has a direct role in regulating cell phenotype, for example, by enforcing CD4 repression that defines the difference between cytotoxic and helper T cell subsets (Sellars et al., 2015). We and others have shown that methylation is tightly coupled to structural features of the genome and that myeloid and lymphoid cells differ substantially in their usage of methylation (Bock et al., 2012; Bröske et al., 2009; Ji et al., 2010; Smith and Meissner, 2013). Localized changes in methylation influence nucleosome stability (Jimenez-Useche et al., 2013) and affect CTCF binding, altering chromatin topology (Flavahan et al., 2016; Ito et al., 2013). From transcription-factor-binding site footprints to nucleosomes, LADs, and X chromosome inactivation, methylation varies among cell types at multiple scales. Given this diversity and the existence of multiple methylation readers and writers (He and Ecker, 2015), the ways in which methylation influences phenotype are likely numerous and pleiotropic.

During hematopoietic development, DNA methylation patterns diverge. Methylation bias toward nucleosome-free DNA increases progressively with T and B lymphocyte development while global methylation levels steadily decline. In contrast, CG methylation appears quite stable during differentiation and activation of both neutrophils and macrophages in the myeloid lineage. Declining global methylation levels in developing lymphocytes likely reflect the emergence of partially methylated domains, which to our knowledge have not been reported in normal cells of the myeloid lineage.

Methylation measurements in leukemias and lymphomas from both lineages are heterogeneous, consistent with the genetic heterogeneity typically observed in cancer samples. The methylation profiles of malignant cells are likely to vary substantially from cell to cell and among samples from different individuals. However, we see general trends here as well. As a group, lymphoid-derived neoplasms continue to lose CG methylation, whereas myeloid cancers have significantly elevated levels of mCH. This observation has potential clinical relevance, as demethylating agents used as chemotherapeutics (Jasielec et al., 2014) could have different effects in myeloid leukemias versus lymphoid malignancies.

The diffuse non-CG methylation we observe is strongly biased toward linker DNA, strongly depleted from LADs, and occurs specifically in all naive T cell samples and the UHPs. Although the spatial enrichment patterns reflecting nucleosome occupancy are similar for mCG and mCH, the occurrence of high levels of diffuse mCH only in specific cell types suggests some degree of independence in the processes governing methylation in CG and non-CG contexts. The cell-type-specific occurrence and association with structural features of the genome further support the interpretation that mCH is a biological phenomenon and not merely a technical artifact.

We showed that mCH levels across samples and cell types are not dependent upon bisulfite conversion rate, given a passably effective bisulfite treatment. Therefore, we suggest that bisulfite conversion efficiency for WGBS studies should be reported based on proper controls using known fully methylated and fully unmethylated DNA and not as the fraction of non-converted non-CG cytosines, as commonly done.

We note that many of the same cell types high in mCH, such as naive T cells, stem cells, and neurons, also have higher reported levels of hydroxymethylation (Booth et al., 2012; Khare et al., 2012; Tsagaratou et al., 2014). As this derivative of methylation also protects cytosines from bisulfite conversion, WGBS does not distinguish methylation from hydroxymethylation, and it is likely that a small fraction of the methylation we report here is due to hydroxymethylation (Yu et al., 2012).

The vast majority of mCH occurs as CA dinucleotides. Deamination of unmethylated cytosine results in uracil, which is efficiently recognized and repaired, but deamination of methylated cytosine (mC) produces thymine. In a mCG dinucleotide context, deamination on one strand creates a T:G mismatch that may be repaired either to the original C:G pair or to a new T:A pair, yielding a mCA dinucleotide on the strand opposite the deamination event. CA dinucleotides resulting from this process (on an evolutionary timescale) may remain susceptible to methylation under certain conditions, such as high local concentration or polymerization of DNA methyltransferases (Rajavelu et al., 2012). This may provide an explanation for the strong bias for CA context in diffuse mCH.

The biochemical basis of non-CG methylation in human DNA is unclear. In mouse embryonic stem cell lines, depletion of either DNMT3A or DNMT3B decreases mCH, whereas depletion of DNMT3L, which does not have a functioning methyltransferase domain, increases mCH levels (Ramsahoye et al., 2000; Tiedemann et al., 2014). In contrast, in germ cells of male mice, mutation of DNMT3L causes severe loss of mCH, which is normally prevalent in this cell type (Ichiyanagi et al., 2013; Vlachogiannis et al., 2015). Possible functional roles for mCH in these contexts remain to be investigated.

The molecular mechanisms of CG methylation have been described using structural and functional studies (Guo et al., 2015; Jia et al., 2007). Although there is substantial overlap in the roles of all three mammalian DNMTs, multiple distinct regulatory pathways have been described for each (Jurkowska et al., 2011). There exist numerous direct and indirect interactions among the readers and writers of histone modifications and DNA methylation, providing physical links and mechanistic explanations for the self-reinforcing loops that appear common in epigenetic regulation (Du et al., 2015).

Limited DNA accessibility due to nucleosome occupancy or association with the nuclear lamina provides a simple explanation of the methylation patterns we observe, both CG and non-CG. Alternatively, DNA methylation may occur in conjunction with nucleosome translocation as part of an active remodeling process that leaves methylated DNA in its wake, as already demonstrated in vitro (Felle et al., 2011). Local DNA methylation patterns may alter the stability of a larger CTCF-nucleosome complex or otherwise contribute to chromatin structure. Furthermore, methyltransferases can remain bound to DNA whether catalytically active or not (Jin et al., 2012; Sharma et al., 2011), raising the possibility of a structural contribution to chromatin, not only for methylated DNA but also for the enzymes responsible for methylation.

Our observation of distinct DNA methylation patterns in different cell lineages parallels the differences seen across the eukaryotic domain. DNA methylation is evolutionarily ancient, with different species developing novel applications. Phylogenetic analysis indicates that the common ancestor of all plants, animals, and fungi possessed a full complement of DNA methylation machinery, including the DNA methyltransferases *DNMT1* and *DNMT3* and a chromomethylase (Zemach et al., 2010). Whereas some species, such as *S. cerevisiae*, have dispensed with DNA methylation, those species that retain methyltransferases commonly share a core set of methylation features, augmented with species-specific variations. For example, gene body methylation (Zemach et al., 2010) and a bias toward linker DNA over nucleosomal DNA appear nearly universal, whereas usage of mCH appears more variable (Huff and Zilberman, 2014). Our results support the view that the maintenance of DNA methylation patterns is fundamentally different between blood-cell lineages and that epigenetic mechanisms may differ substantially between distinct cellular lineages within a multicellular organism as they do among different species.

The functional relevance of specific epigenetic differences among cell types remains to be fully characterized. The large collection of BLUEPRINT WGBS datasets, including several well-defined stages of maturation and additional cell types not discussed here, provides a resource for understanding normal development and a basis for comparison with other cell types and disease states. The accompanying gene expression data and genome-wide maps of histone modifications and DNA accessibility from the same primary samples will aid in these efforts. We expect that a holistic consideration of the diverse components of an epigenome will be necessary for the sensible interpretation of the interdependent epigenetic phenomena directing the expression of our genomic program.

## Experimental Procedures

### Whole-Genome Bisulfite Sequencing

The BLUEPRINT project received ethical review regarding human and animal subjects and genetic data handling. Additionally, approval was obtained at each institute by their respective local ethical review committees. Whole-genome bisulfite sequencing was conducted at the Centre Nacional d’Anàlisi Genòmica as described in Kulis et al. (2015). After cell sorting, genomic DNA libraries were constructed using the Illumina TruSeq Sample Preparation kit (Illumina) following the manufacturer’s standard protocol. DNA was then exposed to two rounds of sodium bisulfite treatment using the EpiTect Bisulfite kit (QIAGEN), and paired-end DNA sequencing was performed using the Illumina Hi-Seq 2000.

We used the GEM mapper (Marco-Sola et al., 2012) with two modified versions each of the human (GRCh37) and viral reference genomes: one with all C’s changed to T’s and another with all G’s changed to A’s. Reads were fully converted in silico prior to mapping to the modified reference genomes, and the original reads were restored after mapping. The first few bases from each read have been shown to have a slightly higher probability of being called as methylated (Schultz et al., 2015), so we trimmed the first ten bases from each read. Heterozygous positions, positions with a genotype error probability greater than 0.01, and positions with a read depth greater than 250 were filtered out. Only cytosines with six or more reads informative for methylation status were considered. On average, half of the reads from either strand will be informative for methylation status at a given position, so minimum coverage is typically greater than 12.

Methylated and unmethylated cytosine conversion rates were determined from spiked-in bacteriophage DNA (fully methylated phage T7 and unmethylated phage lambda). Five samples were excluded based on conversion rates <0.997, supported by visual inspection of CG and non-CG methylation plots. The over-conversion rates for all samples based on methylated phage T7 DNA were ∼5%.

Methylated non-CGs were defined as those cytosines with at least two unconverted reads and a methylation probability greater than two SDs, where the probability of the following base being a guanine was at least two orders of magnitude smaller than the most probable base. The fraction of mCH for each sample is calculated as the number of methylated non-CG cytosines divided by the total number of non-CG cytosines with six or more reads informative for methylation status.

### mCH Spike Detection

Spikes are called in mCH data where the mean methylation value in a sliding window of ten non-CG cytosines exceeds 20%. Only non-CGs with six or more reads informative for methylation are considered.

### Additional WGBS Data

Additional WGBS data for stem cell lines H1 and H9 were generated by the Roadmap Epigenome Project (Lister et al., 2009). WGBS data for stem cell samples (HUES64 cell line) were downloaded from the GEO, accession numbers GSM1112840 and GSM1112841. WGBS data for normal and EBV-transformed B lymphocytes were previously published (Hansen et al., 2014).

### Differential Methylation Analysis

Pairwise sample comparisons were made to determine which sites underwent significant changes in methylation level at each step of B lymphocyte development. All positions that passed quality filtering and were called as homozygous CGs in both samples were evaluated. Significance in the difference between samples in the ratio of converted to unconverted reads at each CG was assessed using Fisher’s exact test if any expected values of the contingency table were less than ten. Otherwise, a chi-square test was used. Results were adjusted for multiple comparisons using the false discovery rate method (Benjamini and Yekutieli, 2001).

### Measuring Oscillation Amplitude

To facilitate high-throughput comparison of many samples, an automated procedure was developed to measure the amplitude of the oscillating methylation signal. To measure amplitude, we must first determine the distance from the CTCF-binding motif of the first peak and the first valley in the methylation signal. Both the period of oscillation and the average methylation levels differ among samples and cell types, so measuring amplitude assuming a fixed period or by using the difference between the maximum and minimum methylation values within a specific distance are unlikely to give good results. Our signal is further complicated by noise and periodic components at multiple scales. Wavelet-based transformation is commonly used in digital signal processing to smooth noisy data and to identify and localize periodic components, making it ideal for this purpose.

A multi-resolution analysis (Percival and Walden, 2006) using the Daubechies wavelet transform (WT) filter of length 16 was used to separate the methylation signal into two distinct components: the average trend in the signal (a wide “V” with lower methylation levels immediately surrounding the occupied CTCF site, gradually increasing to genome-wide average methylation levels within 1 or 2 kb) and the details containing the oscillatory component of the signal at nucleosome scale (Figure S5). Peak and valley positions relative to the CTCF motif were determined using this transformed detail signal. Amplitudes were computed from the original (untransformed) data using the difference in averages of the 10 bp surrounding the WT-identified peak and valley to smooth out local fluctuations (specifically the known 8–10 bp periodicity in methylation levels; Lister et al., 2009). The wavelet transformation was implemented using the R “wavelets” package (Percival and Walden, 2006; R Development Core Team, 2014).

Although we refer to oscillation amplitude in terms of nucleosome influence on methylation, we note that it is determined based only on the methylation signal and does not consider explicit information about nucleosome position.

### Nucleosome Positioning Data

Nucleosome positions for ENCODE cell lines GM12878 (lymphoblastoid) and K562 (myeloid) were inferred from MNase sequencing reads, normalized to input read levels described in Valouev et al. (2011), and downloaded from the University of California, Santa Cruz (UCSC) genome browser. Nucleosome positions for BLUEPRINT samples derived from six histone modification ChIP-seq experiments using NucHunter (Mammana et al., 2013) were downloaded from the BLUEPRINT data portal. DNA methylation information was not used in the comparison of nucleosome localization.

### Visualization

Whole-genome DNA methylation and nucleosome positioning data were aligned to CTCF-binding motifs and plotted for visual inspection. To preserve binding orientation information for CTCF, data surrounding motifs that occurred on the minus strand were reversed. Non-CG methylation levels were computed separately for the strand containing the CTCF-binding motif and the opposite strand. Methylation levels for the opposite strand are plotted as negative values to facilitate visual analysis.

### CTCF-Binding Sites

Occupied CTCF-binding sites were identified by Li et al. (2013) by scanning ChIP-seq peaks using a position-specific scoring matrix for CTCF motifs. Peaks that were detected in at least 95% of ENCODE cell lines were defined as constitutively occupied (cCTCF). We defined lymphoblastoid-specific sites as those present in the GM12878 (lymphoblastoid) cell line, but not in the H1 (human embryonic stem cell) or Nhek (epidermal keratinocyte) cell lines. Stem-cell-specific and skin-cell-specific sites were similarly defined. As a negative control, unoccupied sites were defined as those specifically occupied in another cell type.

### Data Availability

Data generated by the BLUEPRINT project are available through several channels, including genome browsers, Biomart, and directly through the BLUEPRINT portal. Links to data sources are available at the BLUEPRINT website: http://www.blueprint-epigenome.eu.

## Supplemental Information

Supplemental Information includes six figures and one table and can be found with this article online at http://dx.doi.org/10.1016/j.celrep.2016.10.054.

Supplemental information
