# Supplementary material for: Distinct Trends of DNA Methylation Patterning in the Innate and Adaptive Immune Systems
Source: Cell Rep. Author manuscript; Available in PMC 2018 Apr 6. (PMC5889099; doi:10.1016/j.celrep.2016.10.054)
Supplement: Supplemental information [file NIHMS76881-supplement-Supplemental_information.pdf]

Cell Reports, Volume 17

Supplemental Information

Distinct Trends of DNA Methylation Patterning in the Innate and Adaptive Immune Systems

Ronald P. Schuyler, Angelika Merkel, Emanuele Raineri, Lucia Altucci, Edo Vellenga, Joost H.A. Martens, Farzin Pourfarzad, Taco W. Kuijpers, Frances Burden, Samantha Farrow, Kate Downes, Willem H. Ouwehand, Laura Clarke, Avik Datta, Ernesto Lowy, Paul Flicek, Mattia Frontini, Hendrik G. Stunnenberg, José I. Martín-Subero, Ivo Gut, and Simon Heath

SUPPLEMENTAL FIGURES AND TABLE

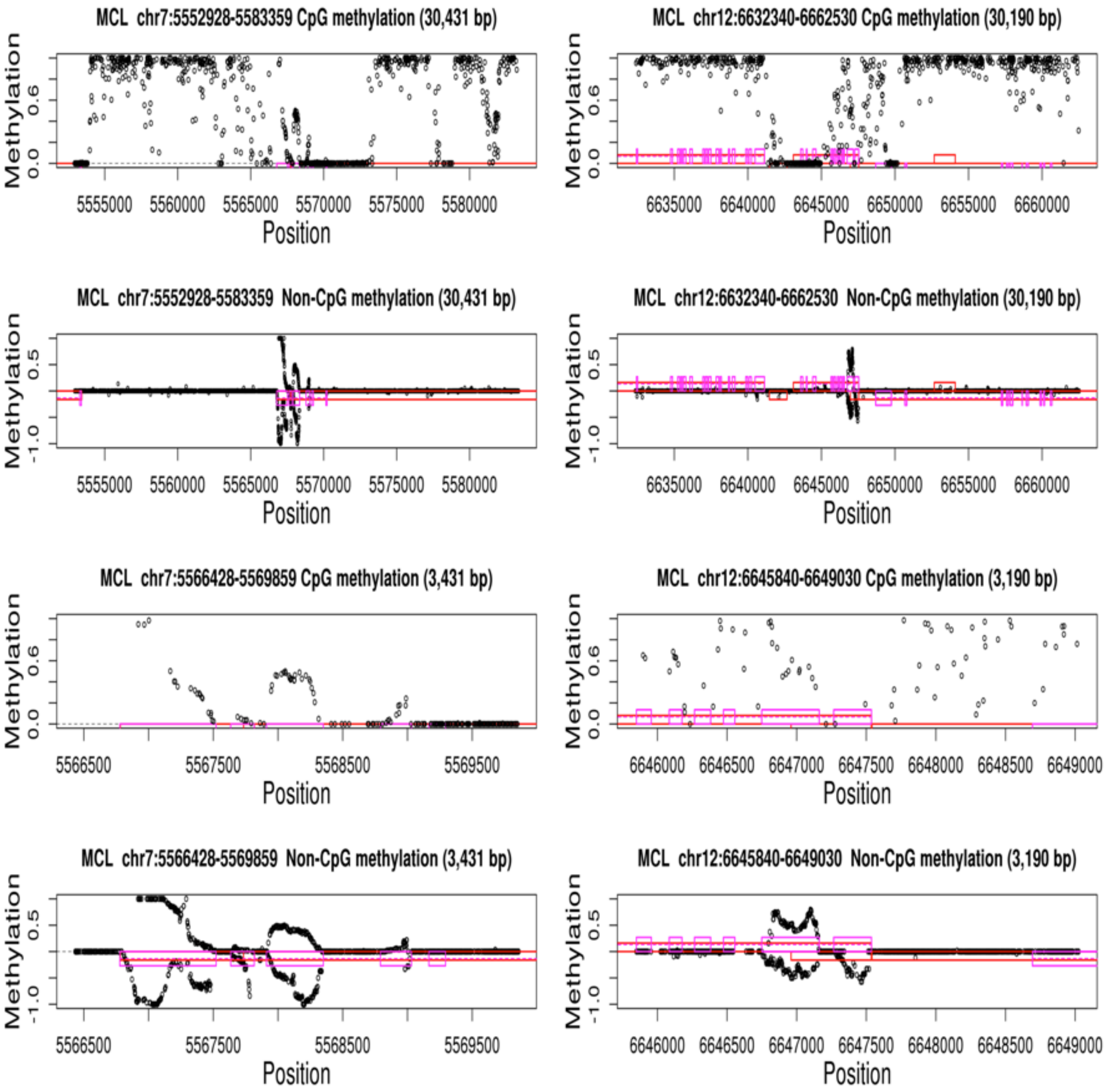

Figure S1. Related to Figure 3. Examples of mCH-marked exons at transitions in CG methylation level. Two examples are shown (left and right columns), each at two resolutions (30k bp and 3.4k bp) to show the mCG transition and the exon specificity, respectively. Each mCG plot is directly above the corresponding mCH plot, as indicated in the figure. Negative values indicate mCH on the minus strand. Exons are shown as blue boxes, above (plus strand) or below (minus strand) the line y=0.

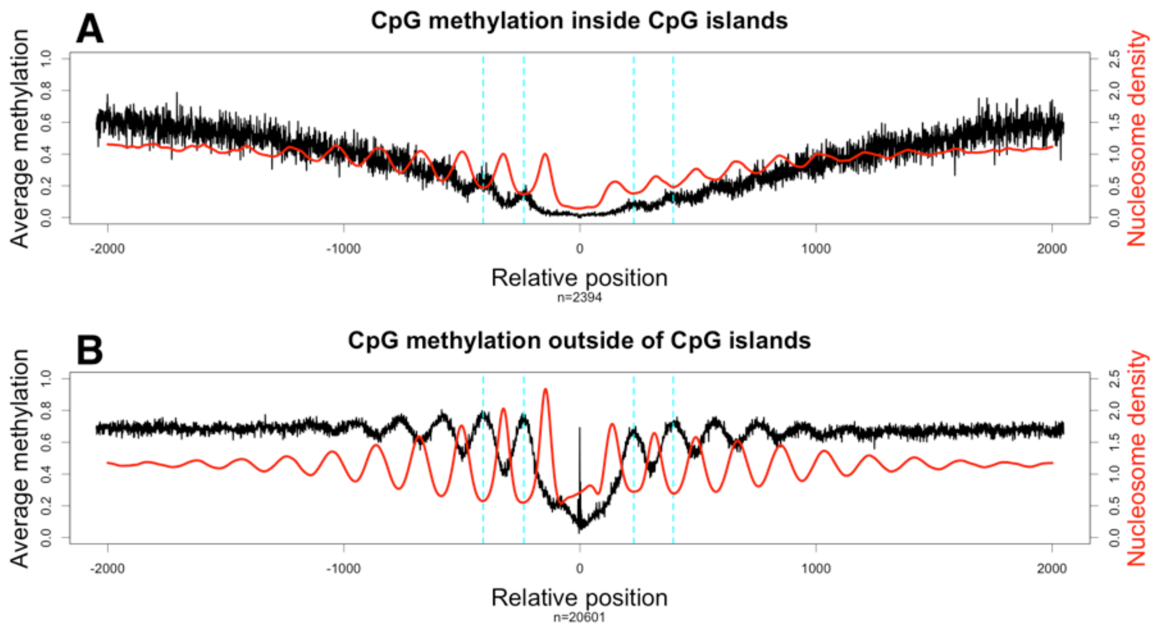

Figure S2. Related to Figure 4. Methylation and nucleosome occupancy at cCTCF binding sites (A) within (n=2394) and (B) outside (n=20601) of CpG islands. Amplitude of both signals is lower within CpG islands, but the phase is the same (dashed vertical lines). Methylation in a plasma cell, nucleosome density in lymphoid cell line GM12878.

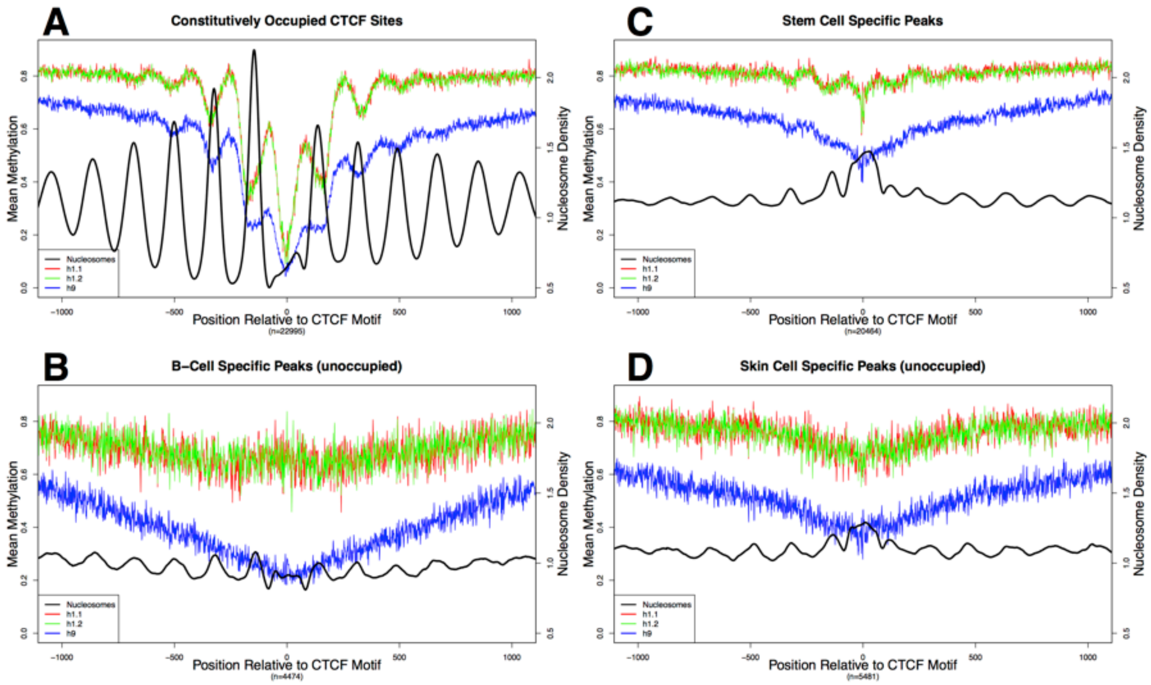

Figure S3. Related to Figure 4. DNA methylation levels in stem cell lines H1 (two replicates; red and green) and H9 (blue) at CTCF binding sites. (A) Constitutively occupied (cCTCF), specifically occupied in (B) lymphoblastoid cell line (unoccupied in stem cells), (C) stem cell line (occupied), (D) skin cell line (unoccupied in stem cells). Nucleosome density (black) from lymphoblastiod cell line.

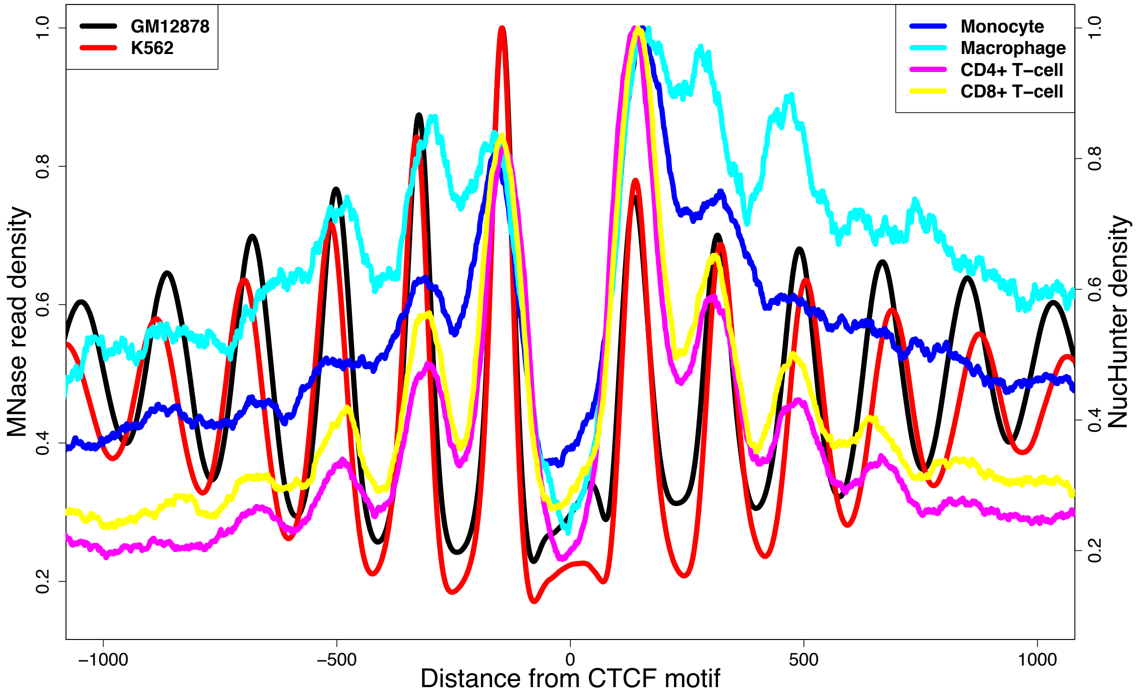

Figure S4. Related to Figure 4A . Nucleosome positioning at constitutively occupied CTCF sites. Positions derived from histone ChIP-seq in Blueprint primary samples (monocyte, blue; macrophage, aqua; and naïve T cells ($CD4^+$, pink; $CD8^+$, yellow)) are spatially consistent with those derived from MNase data in ENCODE cell lines (GM12878, lymphoid, black; and K562, myeloid, red).

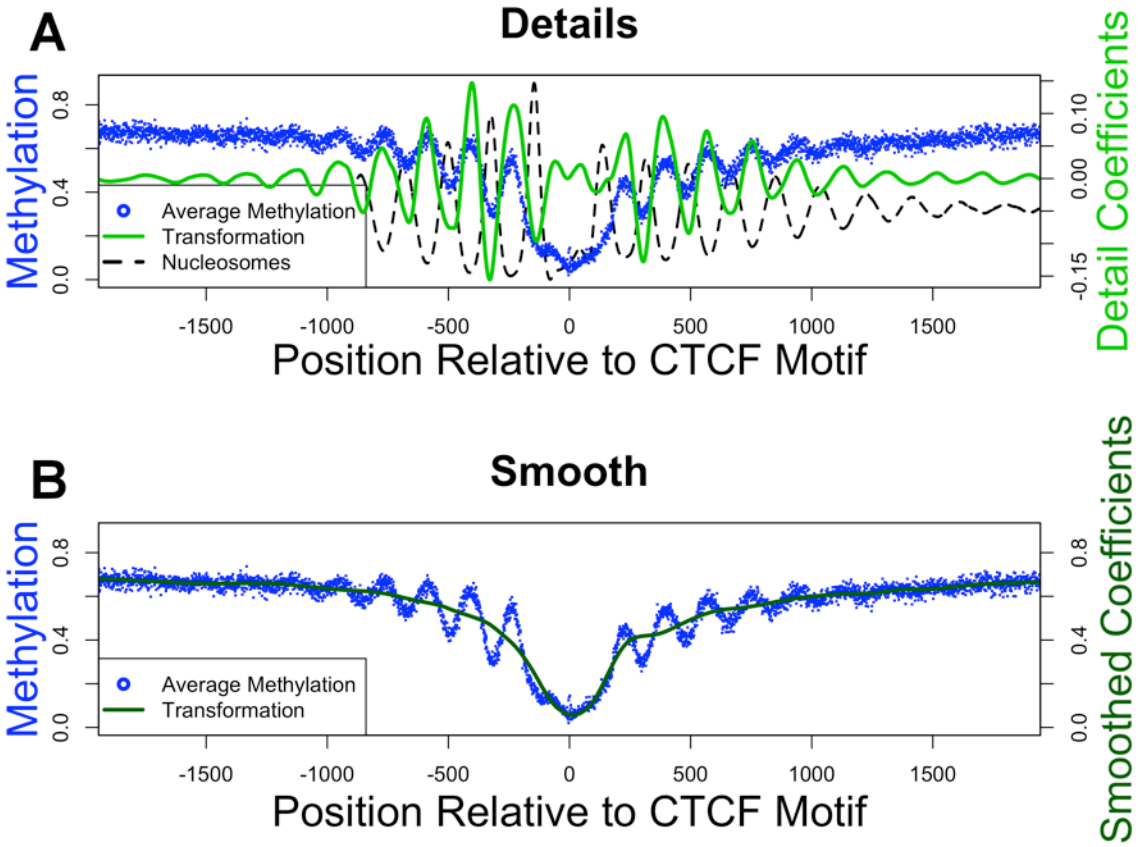

Figure S5. Related to Figure 6. Measurement of nucleosome influence on DNA methylation (oscillation amplitude). The methylation signal (blue) is split into distinct components (green). (A) Maximum and minimum positions relative to the CTCF motif are determined from the wavelet transformation details (light green). Oscillation amplitude is determined from the original (untransformed) signal in a 10 bp window at these two points. (B) Smoothed component of the signal (dark green) reflects low methylation levels near CTCF. Black dashed line: nucleosome density.

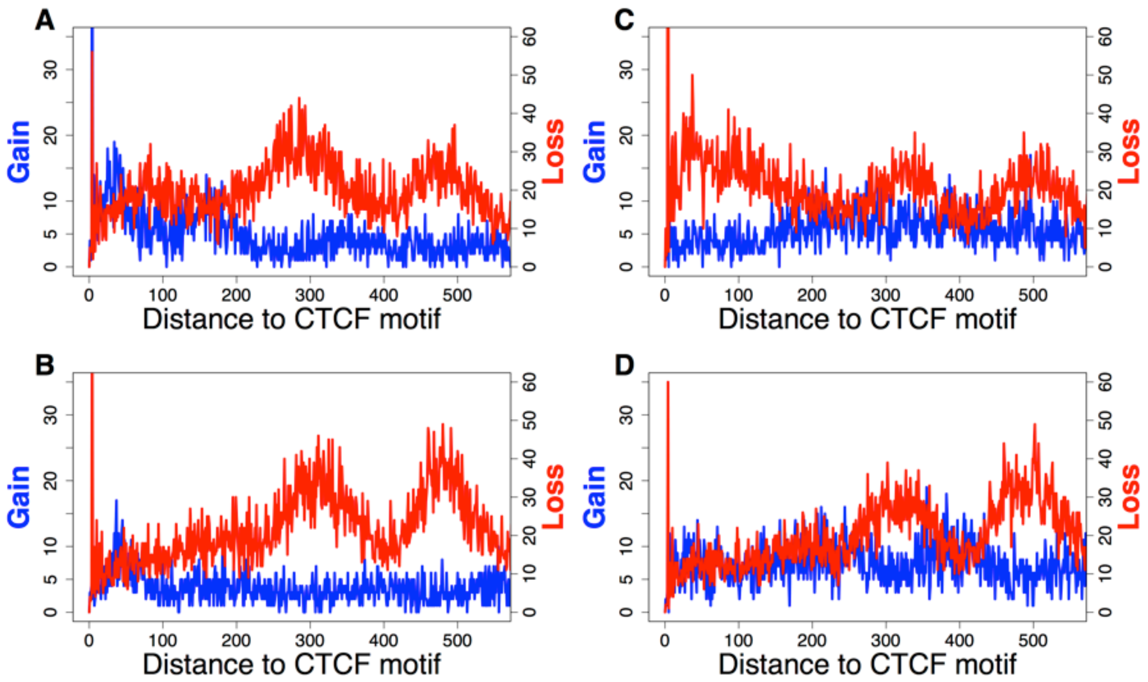

Figure S6. Related to Figures 4A and 6A. Gain (blue) and loss (red) of CG methylation between lymphocyte developmental stages occur in counterphase, reflecting nucleosome positions. Counts of statistically significant changes in methylation at CGs (chi-square or Fisher's exact test) are plotted as a function of distance to the nearest CTCF binding site for each comparison of B cell stages. (A) Pre-B to naïve B cell. (B) Naïve B cell to germinal center B cell. (C) Germinal center B cell to memory B cell. (D) Memory B cell to plasma cell.

Table S1. Cancer sample source material.

Sample ID	Disease	Type of cell	Source	Purification	Karyotype	Purity of tumor cells	Reference
CLL-3	Chronic lymphocytic leukemia	Neoplastic B cell	Peripheral Blood	FACS (CD19+)		>95%	Kulis et al., Nat Genet 2012, PMID: 23064414
CLL-16	Chronic lymphocytic leukemia	Neoplastic B cell	Peripheral Blood	FACS (CD19+)		>95%	Kulis et al., Nat Genet 2012, PMID: 23064414
MM-35574	Multiple myeloma	Neoplastic plasma cell	Bone marrow	FACS (CD138+)		>90%	Agirre et al., Genome Res 2015, PMID: 26437030
MM-35174	Multiple myeloma	Neoplastic plasma cell	Bone marrow	FACS (CD138+)		>90%	Agirre et al., Genome Res 2015, PMID: 26437030
MCL-568	Mantle cell lymphoma	Neoplastic B cell	Lymph node	Not purified		>90%	Queiros et al., submitted
MCL-828	Mantle cell lymphoma	Neoplastic B cell	Peripheral Blood	FACS (CD19+)		>90%	Queiros et al., submitted
S004XMA1	Acute promyelocytic leukemia	Neoplastic myeloid cell	Bone marrow	Not purified	t(15;17)		Petraglia et al., submitted
S0050AA1	Acute promyelocytic leukemia	Neoplastic myeloid cell	Bone marrow	Not purified, ex vivo ATRA treated	t(15;17)		Petraglia et al., submitted
S005FHA1	Acute myeloid leukemia	Neoplastic myeloid cell	Bone marrow	FACS (CD33+)	NK		Yi et al., submitted
S008QKA1	Acute promyelocytic leukemia	Neoplastic myeloid cell	Bone marrow	Not purified	t(15;17)		Petraglia et al., submitted
S008X6A1	Acute myeloid leukemia	Neoplastic myeloid cell	Bone marrow	Not purified	NK		Yi et al., submitted
S00CWTA1	Acute myeloid leukemia	Neoplastic myeloid cell	Venous blood	FACS (CD33+)	NK		Yi et al., submitted
S00CXRA1	Acute myeloid leukemia	Neoplastic myeloid cell	Bone marrow	FACS (CD33+)	NK		Yi et al., submitted
S00CYPA1	Acute myeloid leukemia	Neoplastic myeloid cell	Bone marrow	FACS (CD33+)	NK		Yi et al., submitted
S00D1DA1	Acute myeloid leukemia	Neoplastic myeloid cell	Venous blood	FACS (CD33+)	NK		Yi et al., submitted
S00D39A1	Acute myeloid leukemia	Neoplastic myeloid cell	Venous blood	FACS (CD33+)	NK		Yi et al., submitted
